# Factors associated with development of gastrointestinal problems in patients with scleroderma: a systematic review

**DOI:** 10.1186/s13643-015-0176-2

**Published:** 2015-12-30

**Authors:** Brian Younho Hong, Raymond Giang, Lawrence Mbuagbaw, Maggie Larche, Lehana Thabane

**Affiliations:** Bachelor of Health Sciences (Honours) Program, McMaster University, Hamilton, ON Canada; Statistics for Health (Honours), University of Waterloo, Waterloo, ON Canada; Department of Clinical Epidemiology and Biostatistics, McMaster University, Hamilton, ON Canada; Biostatistics Unit, Father Sean O’Sullivan Research Centre, St. Joseph’s Healthcare, 50 Charlton Avenue East, 3rd Floor Martha Wing, Room H325, Hamilton, ON L8N 4A6 Canada; Centre for Development of Best Practices in Health, Yaoundé Central Hospital, Yaoundé, Cameroon; Division of Rheumatology, Departments of Medicine and Paediatrics, St. Joseph’s Healthcare, McMaster Children’s Hospital, McMaster University, Hamilton, ON Canada; Departments of Paediatrics and Anaesthesia, McMaster University, Hamilton, ON Canada; Centre for Evaluation of Medicine, St. Joseph’s Healthcare, Hamilton, ON Canada; Population Health Research Institute, Hamilton Health Sciences, Hamilton, ON Canada

**Keywords:** Systematic review, Scleroderma, Gastrointestinal, Risk factors

## Abstract

**Background:**

Up to 90 % of people with scleroderma have gastrointestinal (GI) problems such as constipation, bloating, diarrhea, and malabsorption. These problems significantly impair quality of life. Our objective was to determine the risk factors for gastrointestinal issues in people with scleroderma.

**Methods:**

We conducted a systematic review of observational studies that report GI problems in patients with scleroderma along with the associated risk factors. We were interested in any GI problem and any risk factor as long as the study included patients diagnosed with scleroderma according to the 1980 or 2013 American College of Rheumatology guideline. We searched the following databases: CINAHL, EMBASE, LILACS, MEDLINE, and Web of Science for relevant articles from June 1884 to May 2014. Two authors independently screened citations and full text articles and extracted data. Discrepancies were resolved by consensus or by consulting a third author. Methodological quality of included studies was assessed using the Newcastle-Ottawa Scale.

**Results:**

After removing duplicates, 645 unique citations were identified. A total of three studies, three cross-sectional (*n* = 64, *n* = 42, *n* = 606), were included in this systematic review. Collectively, these three studies explored *Helicobacter pylori* and smoking status as risk factors. We found conflicting evidence on the role of *H. pylori* with two studies showing opposite yet statistically significant results. One moderate quality study showed smoking as a risk factor. Key limitations include the small sample sizes of two studies and poor study designs to draw causal links.

**Conclusions:**

There is insufficient evidence to describe the risk factors for GI problems in patients with scleroderma. Longitudinal observational studies are warranted in patients with scleroderma.

**Systematic review registration:**

PROSPERO CRD42014010707

## Background

Scleroderma, otherwise known as systemic sclerosis, is a disease manifested by collagen overproduction affecting various organs including the gastrointestinal (GI) tract [1]. Patients with scleroderma commonly present with inflammation and fibrosis of the skin, vascular abnormalities, organ damage, and autoantibody production [2]. Diagnosis is made when these signs and corresponding symptoms are present [3]. Scleroderma is a debilitating disease that is estimated to affect approximately 16,000 Canadians—with four to five times more females than males [1]. The prevalence of the disease is higher among people of African origin and varies across countries, appearing more in North America and Australia than in Europe and Japan [4]. Scleroderma is associated with debilitating morbidity such as reduction in mobility and depression [5]. Patients with this disorder have a 30.8 % mortality rate, although this number can vary depending on gender and which visceral organs are involved [6]. Currently, there are no effective treatments to combat scleroderma, partly because its pathogenesis remains unclear [7]. Consequently, much attention has shifted towards predicting the complications of scleroderma and managing them appropriately [8]. The underlying rationale is that it is easier to manage the disease before these complications arise [8].

In around 90 % of all patients with scleroderma, the GI tract is involved. This contributes considerably to impairment in quality of life [9–11]. Malabsorption, gastroesophageal reflux, nausea, vomiting, diarrhea, and constipation are some of the GI complications that can occur [9]. Although environmental risk factors are clearly linked to increased risk of developing scleroderma [12], it is unclear whether the GI involvement secondary to scleroderma is influenced by these environmental factors [12]. This study reviews the association between GI involvement and environmental and other exposures in patients with scleroderma. These findings will help patients with scleroderma and healthcare professionals in preventing GI morbidity. In Canada, the average annual economic burden of scleroderma is estimated to be $18,453 per patient [13]. Knowing that the cost of care for patients with scleroderma increases with more organ involvement, the findings from this study can inform policy developers to identify ways to curb healthcare costs [13]. The objective of this systematic review was to determine the factors associated with development of GI problems in patients with scleroderma.

## Methods

### Criteria for inclusion of studies

#### Design and time frame

Observational studies from June 1884 to May 2014 were considered in this review. June 1884 was chosen because it is the earliest entry of scleroderma in available databases.

#### Exposure

The exposures of interest were silica, silicone implantation and rupture, antacid, solvents, epoxy resins, welding fumes, anorexigens, pentazocine, bromocriptine, l-tryptophan, pesticides, constant hand/arm vibration, alcohol, intestinal microbiota, and food along with any other reported risk factors [14–19].

#### Participants

The population of interest was patients with a formal diagnosis of systemic sclerosis according to the 1980 and 2013 American College of Rheumatology guidelines [20, 21].

#### Outcome

Outcomes of interest included any reported GI problems such as nausea, vomiting, and diarrhea.

### Search methods for identification of studies

#### Search strategy

The lead author (BYH) with the help of a hospital librarian (LC) developed the search strategy. We designed a comprehensive and exhaustive search strategy of five electronic databases to identify observational studies that reported on risk factors for GI problems in scleroderma. Our search strategy for MEDLINE via Ovid is reported in Table 1.

**Table 1:** Search strategy of MEDLINE via Ovid

	Database: Ovid MEDLINE(R)
	Search strategy:
1.	scleroderma.mp.
2.	systemic sclerosis.mp.
3.	exp scleroderma, systemic/
4.	exp scleroderma, localized/
5.	or/1-4
6	risk.mp.
7.	exp risk/
8.	((factor$ or feature$ or aspect$ or characteristic$) adj4 (risk)).mp.
9.	exp silicon dioxide/
10.	exp silicones/
11.	exp antacids/
12.	exp solvents/
13.	organic solvent$.mp.
14.	exp epoxy resins/
15.	welding fume$ or welding gas*.mp.
16.	exp anorexigenic drugs/
17.	exp pentazocine/
18.	exp bromocriptine/
19.	exp tryptophan/
20.	exp pesticides/
21.	exp vibration/
22.	hand vibration or arm vibration.mp.
23.	exp alcohol drinking/
24.	or/6-23
25.	exp gastrointestinal tract/
26.	exp gastrointestinal diseases/
27.	((problem$ or issue$ or concern$ or complication$) adj3 (alimentary canal or alimentary tract or digestive tube or digestive tract or GI tract)).mp.
28.	exp weight loss/
29.	uninten** weight loss.mp.
30.	((uninten**) adj3 (weight loss)).mp.
31.	exp appetite/
32.	poor appetite or limited appetite or reduced appetite or sparse appetite.mp. 34. ((poor or limited or reduced or sparse) adj3 (appetite)).mp.
35.	exp deglutition disorders/
36.	dysphagia or swallowing disorders.mp.
37	exp gastroesophageal reflux/
38.	acid reflux.mp.
39.	exp colonic pseudo-obstruction/
40.	exp duodenal obstruction/
41.	exp intestinal obstruction/
42.	exp intestinal pseudo-obstruction/
43.	chok*.mp.
44.	early satiety.mp.
45.	bloat* or disten* or bulg* or enlarg* or expan*.mp.
46.	exp nausea/
47.	exp vomiting/
48.	exp constipation/
49.	exp diarrhea/
50.	((use or prescri** or administ**) adj2 (antibiotic$ or antibacterial$ or antimicrobial$)).mp.
51.	exp steatorrhea/
52.	exp fecal incontinence/
53.	exp parenteral nutrition/
54.	or/25-53
55.	5 and 24 and 54

#### Databases searched

Cumulative Index to Nursing and Allied Health Literature (CINAHL), Excerpta Medica database (EMBASE), Latin American and Caribbean Health Sciences Literature (LILACS), Medical Literature Analysis and Retrieval System Online (MEDLINE), and Web of Science were searched to identify relevant citations for this systematic review.

#### Other sources searched

We manually checked the reference lists of studies identified in an attempt to identify additional publications. Conference websites were not separately searched because EMBASE catalogues abstracts from several notable rheumatology conferences. A practicing rheumatologist and also an author of this review (ML) was queried to identify additional studies. We searched the Scleroderma Society of Canada, the Scleroderma Foundation, and the Scleroderma Research Foundation websites.

### Study screening, selection, and assessment of methodological quality

Two authors (BYH and RJ), working independently, reviewed all citations and abstracts resulting from the search strategy to identify eligible papers. The full text of eligible articles were obtained and assessed independently using a pre-designed eligibility form based on the inclusion criteria. Eligible studies were included in the review. Disagreements were resolved by discussion or by seeking the opinion of a third party (LT/ML). The two authors (BYH and RJ) independently assessed the methodological quality of included studies using the Newcastle-Ottawa Scale and extracted data using a pre-established data extraction form [22].

### Data analysis

We planned to pool the results using the DerSimmonian and Laird random effects method if there was a measure of relative risk (risk ratio, odds ratio, hazard ratio) and precision (95 % confidence intervals, standard error, variance) and if there was sufficient clinical and statistical homogeneity [23], and present the results as an odds ratios with 95 % confidence interval and *p* values. Between-study heterogeneity was also to be tested using Cochran’s Q and the *I*^2^ statistic. However, given the considerable clinical and design heterogeneity in the included studies, we adopted a narrative approach to summarize our findings.

## Results

### Results of search

**Fig. 1:**
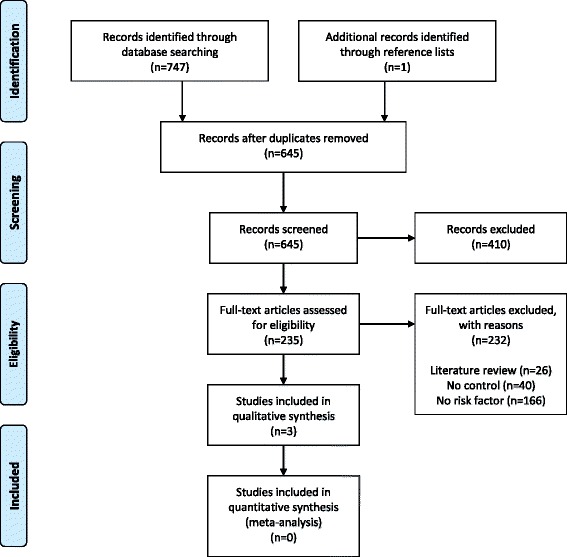
Flow diagram of study selection

Our electronic literature search identified a total of 645 unique citations after duplicates were removed. One study was identified by searching the reference lists of included studies, but was excluded from this review. Title and abstract screening identified 235 articles that were potentially eligible for inclusion. During the full text screening, 232 citations were removed resulting in a total of three studies being included [24–26]. Figure 1 provides a flow diagram of the study selection process and the reasons for exclusion.

### Overview of study characteristics and summary of study findings

There were three studies included in this systematic review, conducted in Japan, Canada, and Croatia in 2008, 2011, and 2013, respectively [24–26]. The number of enrolled patients ranged from 42 to 606 with the total of 712 patients included in this systematic review [24–26]. All three citations were cross-sectional studies [24–26]. Although all three studies examined GI problems as the outcome of interest, only one used endoscopic examination to diagnose reflux esophagitis [24], while the other two relied on self-reported severity of GI problems. Two studies examined *Helicobacter pylori* as a risk factor for GI problems in patients with scleroderma but gave contrasting results [24, 26]. The three included studies are summarised in Table 3.

#### Yamaguchi et al. [24]

The study by Yamaguchi et al. was a cross-sectional study that examined whether or not the presence of *H. pylori* infection was associated with reflux esophagitis in patients with scleroderma. Of the 138 patients with scleroderma, 74 were excluded because they were taking medications for their GI issues or had laparotomy. Of the remaining 64 patients (mean age = 60.7 years, % female 81.3, % diffuse disease 17), 37 were *H. pylori* positive. Without adjusting for covariates, it was found that being *H. pylori* positive is protective against reflux esophagitis in patients with scleroderma (OR = 0.16, 95 % CI = 0.05–0.47).

#### Hudson et al. [25]

The study by Hudson et al. was also a cross-sectional study, which examined whether smoking (never, past, or current) was associated with GI problems in patients with scleroderma. It examined the presence of 14 different GI problems and the severity of GI symptoms. Six hundred and six patients (mean age = 55.3 years, % female 87, % diffuse disease 36) were included in the analysis. Adjusted for covariates (age, gender, ethnicity, disease duration, diffuse disease), the regression model showed that only the number of GI symptoms and poor appetite showed statistical significant worsening with smoking.

#### Radic et al. [26]

The study by Radic et al. was another cross-sectional study that examined whether or not the presence of *H. pylori* infection was associated with self-reported GI problems in patients with scleroderma. Of the 42 patients (mean age = 54.3 years, % female 90.5, % diffuse disease 95.3), 26 were *H. pylori* positive. An adjusted analysis revealed that being *H. pylori* positive is correlated with high prevalence of GI issues.

### Methodological quality of included studies

Methodological quality of included studies was assessed using the Newcastle-Ottawa Scale and graded according to the guidelines [22]. Being that all three studies were cross-sectional, two criteria regarding follow-up in the Newcastle-Ottawa Scale did not apply. Thus, of the possible 6 points, Yamaguchi et al. scored 4, Radic et al. scored 5, and Hudson et al. scored 6 points. Overall, the quality of included studies were generally low (see Table 2).

**Table 2:** Methodological quality of included studies (Newcastle-Ottawa Scale)

Author, year	Selection				Comparability	Outcome		
	Representativeness of the exposed cohort	Selection of the non-exposed cohort	Ascertainment of exposure	Outcome of interest not present at start of study		Assessment of outcome	Adequacy of duration of follow-up	Adequacy of completeness of follow-up
Yamaguchi et al. [24]	√	√	√	‡	‡	√	N/A	N/A
Hudson et al. [25]	√	√	‡	√	√	‡	N/A	N/A
Radic et al. [26]	√	√	√	‡	√	√	N/A	N/A

√ = yes; ‡ = no

## Discussion

### Summary of main findings

A total of three cross-sectional studies generally of low quality were included in this systematic review (Table 3). Two studies explored *H. pylori* infection as a risk factor for GI problems in patients with scleroderma but showed opposing results. Although Yamaguchi et al. [24] had a slightly bigger sample size, only Radic et al. [26] controlled for confounding variables in the analysis. The difference between these two studies may also be attributable to the difference in the patient demographics and outcome adjudication. Only 17 % of the patients had diffuse scleroderma in Yamaguchi et al.’s study, while 95 % had diffuse scleroderma in Radic et al.’s study [26]. Furthermore, Yamaguchi et al. [24] employed endoscopic examination to diagnose reflux esophagitis, while Radic et al. [26] used patient-reported GI problems. Hudson et al.’s study examines the effect of smoking on GI problems in patients with scleroderma and showed that higher proportion of patients with scleroderma who smoke had GI problems [25]. Yamaguchi et al., Hudson et al., and Radic et al. [24–26] looked at reflux esophagitis, poor appetite, and GI symptom severity as the outcome, respectively. None of the papers reviewed discussed other environmental exposures such as silica and epoxy.

**Table 3:** Summary of included studies

Author, year	Country	Time	Study design	Sample size	Age (years)	Male:female	Patient characteristics	Exposure	Outcomes	
Yamaguchi et al. [24]	Japan	Oct 1998–June 2005	Cross-sectional	*n* = 64	Mean, range = 60.7, 24–85	12:52	Disease duration (<1 year, <5, <10, >10): *n* = 19, *n* = 17, *n* = 18, *n* = 10	*H. pylori*: positive (*n* = 37), negative (*n* = 27)	*H. pylori* positive	
									% reflux esophagitis 27	
							% diffuse disease 17		*H. pylori* negative	
							% smoking 30		% reflux esophagitis 70	
							% alcohol 34			
							% corticosteroid use 72			
Hudson et al. [25]	Canada	Aug 2004–Nov 2009	Cross-sectional	*n* = 606	Mean, SD = 55, 12	79:527	% Caucasian: 87.0	Smoking status: never (*n* = 255), past (*n* = 252; mean pack-years of smoking = 17, SD = 18), current (*n* = 99; mean pack-years of smoking = 25, SD = 17)	Never	Current
							Disease duration (mean, SD): 11, 9		Mean severity of GI symptoms: 1.61 out of 10	Mean severity of GI symptoms: 2.18 out of 10
							% diffuse disease 36.0		Mean number of GI symptoms: 3.99 out of 14^a^	Mean number of GI symptoms: 4.91 out of 14^a^
									% poor appetite 28.6^a^	% poor appetite 47.5^a^
									% difficulty swallowing 54.1	% difficulty swallowing 59.6
									% acid reflux 60.8	% acid reflux 68.7
									% nocturnal choking 28.6	% nocturnal choking 29.3
									% heartburn 41.6	% heartburn 54.5
									% early satiety 38.8	% early satiety 51.5
									% abdominal bloating 34.9	% abdominal bloating 43.4
									% nausea and vomiting 14.1	% nausea and vomiting 23.2
									% chronic constipation 25.5	% chronic constipation 32.3
									% chronic diarrhea 23.9	% chronic diarrhea 26.3
									% antibiotics for bacterial overgrowth 7.5	% antibiotics for bacterial overgrowth 6.1
									% greasy stools 18.8	% greasy stools 24.2
									% fecal incontinence 19.2	% fecal incontinence 22.2
									% parenteral nutrition 2.4	% parenteral nutrition 2.0
									% taking medications for GI symptoms 73.7	% taking medications for GI symptoms 59.6
									% taking medications for GI symptoms 67.1	
Radic et al. [26]	Croatia	Jan 2009–Dec 2010	Cross-sectional	*n* = 42	Mean, SD = 54.3, 13.6	4:38	Disease duration (median, range): 6, 1–43 years	*H. pylori*: positive (*n* = 26), negative (*n* = 16)	*H. pylori* positive	
									% GI problems 85	
							% diffuse disease 95.3		*H. pylori* negative	
									% GI problems 31	

^a^Denotes *p* value less than 0.05

### Limitations

There are limitations to be noted in our systematic review. First, while our search did not include the gray literature, we undertook a thorough and rigorous search of CINAHL, EMBASE, GI, LILACS, and MEDLINE. Second, the extent of available data with regards to covariates was limited and the length of follow-up was often not specified, which may not be adequate for the observation of GI problems in patients with scleroderma. Third, all three studies are cross-sectional, which are not useful in determining predictors of outcome. These deficiencies highlight the need for further research to address the current knowledge gap exploring the risk factors associated with GI problems in patients with scleroderma.

### Implications for practice

There is not enough evidence to inform clinical practice. This paper included three studies that report *H. pylori* infection (two studies) and smoking (one study) as potential risk factors for GI problems in patients with scleroderma. Given that the evidence is sparse and generally of low quality and that the two included studies on *H. pylori* contradict each other. It is unclear how to incorporate these findings into clinical practice. Smoking, however, seems to be associated with a wide range of GI problems.

### Implications for research

In the field of scleroderma, longitudinal observational studies are warranted to investigate the risk factors for GI problems. For rare exposures like silica and pesticides, case-control designs may be more appropriate. A wealth of useful information can also be obtained from well-kept scleroderma registries. The studies should include patients with scleroderma followed up prior to and after the development of GI problems. Data on all potential risk factors including *H. pylori* infection, smoking, exposure to environmental toxins, and others should be collected. All possible GI problems should be explored, including data on morbidity and mortality.

## Conclusions

There is a lack of evidence to conclusively suggest or refute whether *H. pylori* infection or smoking is a definitive risk factor for developing GI problems in patients with scleroderma.

## Abbreviations

CINAHL: Cumulative Index to Nursing and Allied Health Literature
EMBASE: Excerpta Medica Database
GI: gastrointestinal
LILACS: Latin American and Caribbean Health Sciences Literature
MEDLINE: Medical Literature Analysis and Retrieval System Online
